# Single-cell analysis at the protein level delineates intracellular signaling dynamic during hematopoiesis

**DOI:** 10.1186/s12915-021-01138-6

**Published:** 2021-09-09

**Authors:** Jinheng Wang, Chenggong Tu, Hui Zhang, Yongliang Huo, Eline Menu, Jinbao Liu

**Affiliations:** 1 Affiliated Cancer Hospital & Institute of Guangzhou Medical University, Guangzhou, 510095 China; 2 Guangzhou Municipal and Guangdong Provincial Key Laboratory of Protein Modification and Degradation, State Key Laboratory of Respiratory Disease, School of Basic Medical Sciences, Guangzhou Medical University, Guangzhou, 511436 China; 3 Department of Hematology and Immunology, Myeloma Center Brussels, Vrije Universiteit Brussel, 1090 Brussels, Belgium

**Keywords:** Mass cytometry, Single-cell analysis, Signaling biosignatures, Hematopoietic stem and progenitor cells, Hematopoiesis, Trajectory

## Abstract

**Background:**

Hematopoietic stem and progenitor cell (HSPC) subsets in mice have previously been studied using cell surface markers, and more recently single-cell technologies. The recent revolution of single-cell analysis is substantially transforming our understanding of hematopoiesis, confirming the substantial heterogeneity of cells composing the hematopoietic system. While dynamic molecular changes at the DNA/RNA level underlying hematopoiesis have been extensively explored, a broad understanding of single-cell heterogeneity in hematopoietic signaling programs and landscapes, studied at protein level and reflecting post-transcriptional processing, is still lacking. Here, we accurately quantified the intracellular levels of 9 phosphorylated and 2 functional proteins at the single-cell level to systemically capture the activation dynamics of 8 signaling pathways, including EGFR, Jak/Stat, NF-κB, MAPK/ERK1/2, MAPK/p38, PI3K/Akt, Wnt, and mTOR pathways, during mouse hematopoiesis using mass cytometry.

**Results:**

With fine-grained analyses of 3.2 million of single hematopoietic stem and progenitor cells (HSPCs), and lineage cells in conjunction with multiparameter cellular phenotyping, we mapped trajectories of signaling programs during HSC differentiation and identified specific signaling biosignatures of cycling HSPC and multiple differentiation routes from stem cells to progenitor and lineage cells. We also investigated the recovery pattern of hematopoietic cell populations, as well as signaling regulation in these populations, during hematopoietic reconstruction. Overall, we found substantial heterogeneity of pathway activation within HSPC subsets, characterized by diverse patterns of signaling.

**Conclusions:**

These comprehensive single-cell data provide a powerful insight into the intracellular signaling-regulated hematopoiesis and lay a solid foundation to dissect the nature of HSC fate decision. Future integration of transcriptomics and proteomics data, as well as functional validation, will be required to verify the heterogeneity in HSPC subsets during HSC differentiation and to identify robust markers to phenotype those HSPC subsets.

**Supplementary Information:**

The online version contains supplementary material available at 10.1186/s12915-021-01138-6.

## Background

Hematopoietic stem cells (HSCs) can self-renew and differentiate into all lineages of mature blood cells and are thus regarded as the source and foundation of hematopoiesis and immune activation. In the dynamic HSC niche, which contains a range of mediators of signaling crosstalk, the type and strength of signaling in HSC is ever changing to maintain the balance between self-renewal and differentiation [1–3]. During development or injury, the fate of HSC relies on intracellular signaling which ultimately determines their differentiation trajectories [4]. It is highly desirable to accurately quantify the dynamic changes of signaling pathways in stem, progenitor, and lineage cells to fully understand the fundamental mechanisms of self-renewal and differentiation. For now, several conserved signaling pathways, such as Wnt [5–8], Notch [9, 10], Hedgehog [11, 12], and TGF-β/SMAD [13, 14] have been well-documented in regulating self-renewal, proliferation, differentiation, senescence, and quiescence of HSC. However, a comprehensive landscape recapitulating the dynamics of major signaling pathways during the differentiation from HSC to all the progenitor and lineage cells has not been completely revealed.

In mice, hematopoietic stem and progenitor cell (HSPC) subsets, including long-term (LT)-HSC, multipotent progenitor (MPP), lymphoid-primed multipotent progenitor (LMPP), common myeloid progenitor (CMP), granulocyte-macrophage progenitor (GMP), and Mgk/erythroid progenitor (MEP), have been typically identified with a combination of phenotypic cell surface markers, including c-Kit, Sca-1, CD16/32, CD34, Flk2, CD150, and CD48 [15]. Although surface marker-based identification has been widely used in HSC research, heterogeneity within these progenitors has been revealed [16]. To resolve this heterogeneity, a series of single-cell technologies that enable us to analyze large numbers of individual cells at the genomic, transcriptomic, proteomic, and epigenetic level have been developed [17–19]. Advanced single-cell RNA sequencing enables the dissection of the extensive degree of heterogeneity in complex cellular systems by simultaneously measuring expression of up to 10,000 genes in a single cell [20, 21]. This powerful tool has been widely employed to elucidate the heterogeneity of the hemopoietic system, to identify the HSC differentiation hierarchy, and to discover underlying novel hematopoietic subsets [22–25]. However, a number of complicated and varied post-transcriptional mechanisms, such as translation rates, translation rate modulation, protein modification, regulation of a protein’s half-life, protein synthesis delay, and protein transport, are involved in turning mRNA into bioactive protein [26–28]. Quantification at transcript levels is not sufficient to predict their protein levels and to explain genotype-phenotype relationships under various scenarios [28], including stem cell self-renewal and differentiation. Thus, demonstrating dynamic transitions of key proteins’ abundance at the single-cell resolution is indispensable for the complete and precise understanding of complex processes in hematopoiesis.

Mass cytometry, a cutting-edge technology merging inductively coupled plasma mass spectrometry and flow cytometry, uses antibodies labeled with rare heavy metal [29, 30] and permits over 40 parameters to be simultaneously determined at the single-cell level in a high-throughput manner with minimal/no compensation and a fine resolution [31, 32]. Such highly multiparametric detection initiates an unprecedented way to accurately quantify the surface and intracellular proteins and promotes the in-depth analysis of cellular heterogeneity, the discovery of new cell subsets, and the identification of putative differentiation routes [33, 34]. Mass cytometry-based single-cell analysis of proteomics has already been used to construct hematopoietic differentiation pathways [31], map cell cycle phases for hematopoietic cells [35], and accurately predict B cell developmental trajectories [36].

To systemically capture the activation dynamics of 8 signaling pathways at the single-cell level during hematopoiesis, we simultaneously evaluated 11 key proteins in these pathways in millions of bone marrow (BM) cells at different stages of hematopoiesis. In tandem with highly multivariate cellular phenotyping, we used mass cytometry to examine the activation status of the EGFR, Jak/Stat, NF-κB, MAPK, PI3K/Akt, Wnt, and mTOR pathways in 22 HSPC and lineage subsets. We systematically assessed the changes of signaling proteins in HSPC and lineage subsets and revealed their transition patterns in all these subsets during hematopoiesis. Advanced single-cell analysis of pathway activation status within surface marker-defined HSPC subsets made it possible to map a trajectory of signaling programs in HSC differentiation. We observed distinct signaling programs in differentiation routes from LT-HSC to progenitor and lineage cells, and demonstrated links between HSPCs and lineage cells, as well as between activation statuses of signaling pathways during hematopoiesis. Using these mass cytometry data, we provide substantial information regarding the regulation of signaling pathways in HSC differentiation and hematopoietic recovery, thus offering a powerful new level of insight into the hematopoiesis regulation and laying a solid foundation to dissect the nature of HSC fate decision.

## Results

### An atlas of signaling profiles in HSPC

**Fig. 1:**
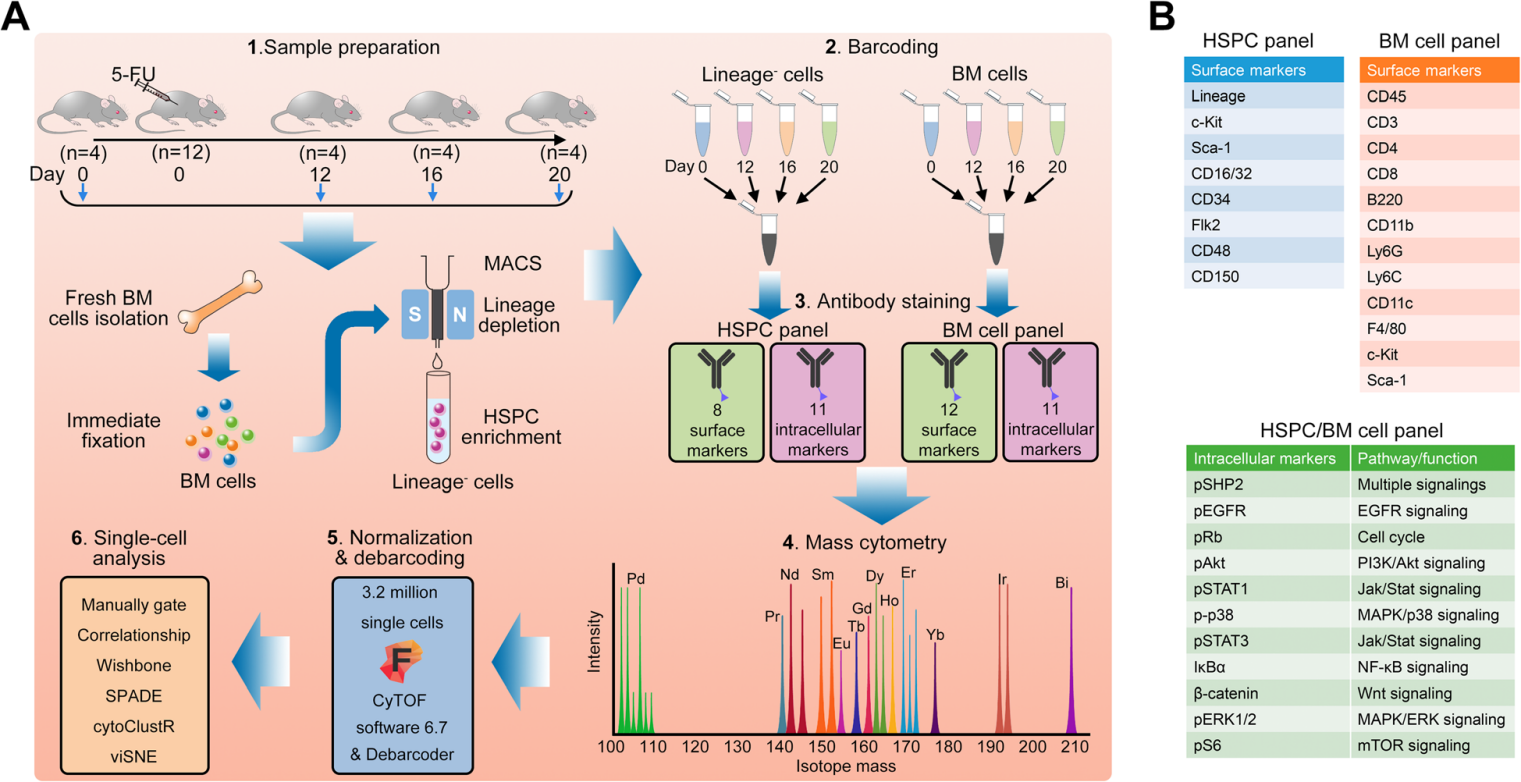
Characterizing the signal pattern of hematopoietic cells in hematopoiesis. **A** Experimental workflow and approaches used in this study. **B** Markers and proteins used to characterize the signaling atlas of HSPC and BM cells

To comprehensively profile signaling programs in mouse hematopoiesis at the protein level, we implemented a mass cytometry-based high-dimensional single-cell analysis (Fig. 1a). For retaining the maximum signaling information, the BM cells were fixed immediately after isolation from mice. HSPC were enriched through lineage depletion using MACS, thus ensuring the sufficiency of cells for single-cell analysis. Enriched HSPC and BM cells were barcoded and stained separately with two antibody cocktails recognizing surface markers and intracellular signaling proteins (Fig. 1b). Millions of cells were systematically analyzed using advanced methods developed for mass cytometry-based single-cell analyses.

**Fig. 2:**
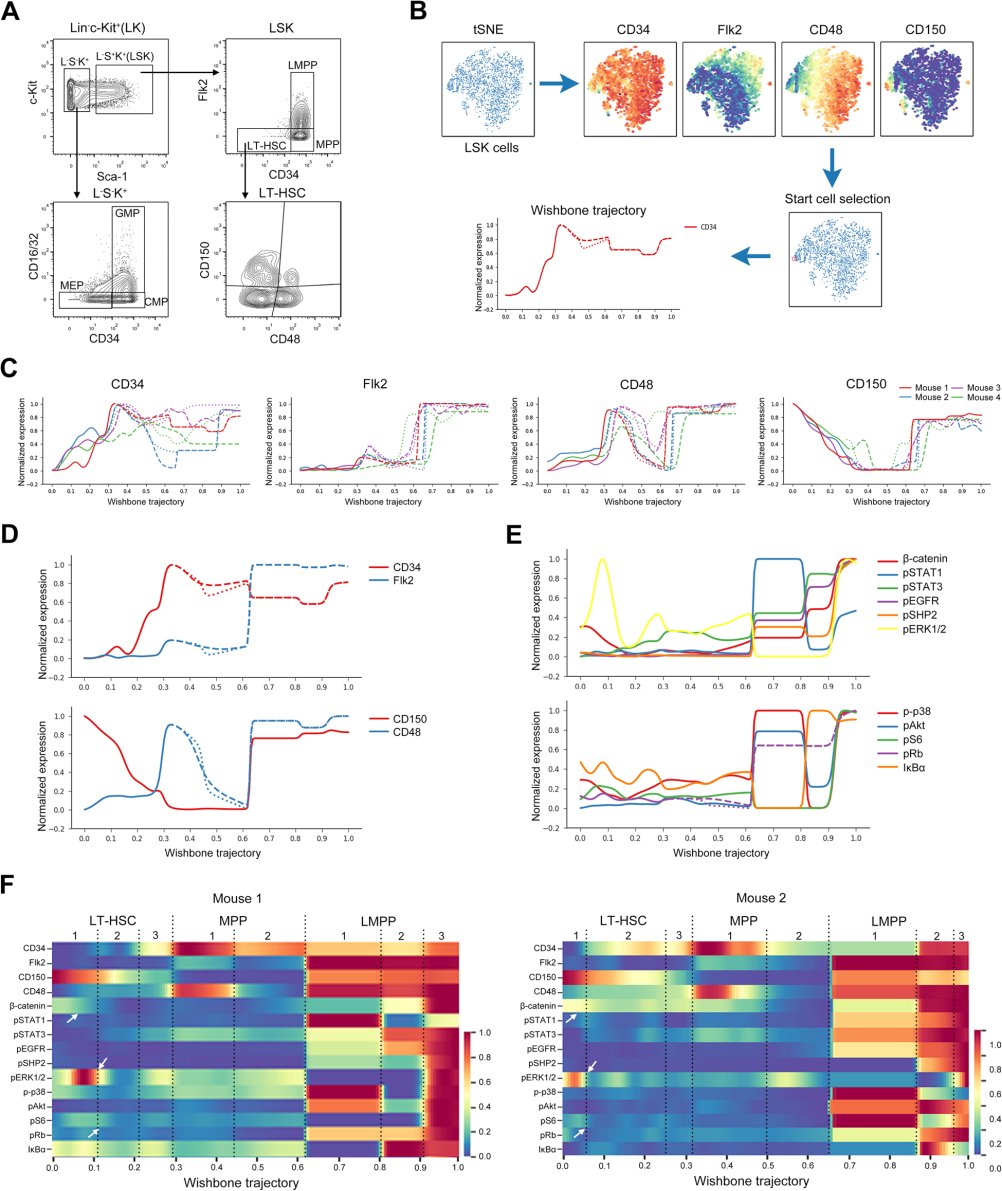
Wishbone trajectory of HSC differentiation. **A** Gating for HSPC subsets. **B** An approach for Wishbone analysis. **C** Wishbone trajectory showing the dynamics of CD34, Flk2, CD48, and CD150 in BM LSK cells from 4 mice. **D** Wishbone trajectory showing the dynamics of CD34, Flk2, CD48, and CD150 in LSK cells (*n* = 2296) from one representative mouse. **E** Plots comparing the dynamics of the indicated proteins along the trajectory in LSK cells. **F** Derivative plot showing the changes in expression of the indicated proteins along the trajectory in mouse 1 (*n* = 2296 LSK cells) and 2 (*n =* 4950 LSK cells). HSPC subsets and stages were divided using dotted lines. Arrows indicate the main signaling proteins in LT-HSC. Color bars indicate the normalized expression level

After measurement using mass cytometry, several HSPC subsets, including Lin^−^Sca-1^−^c-Kit^+^ (L^−^S^−^K^+^), Lin^−^Sca-1^+^c-Kit^+^ (LSK), LT-HSC (CD34^−^Flk2^−^LSK), MPP (CD34^+^Flk2^−^LSK), LMPP (CD34^+^Flk2^+^LSK), CMP (CD16/32^−^CD34^+^L^−^S^−^K^+^), GMP (CD16/32^+^CD34^+^L^−^S^−^K^+^), MEP (CD16/32^−^CD34^−^L^−^S^−^K^+^), were gated [15] (Fig. 2a). Pseudotime and trajectory analysis are frequently used for recovering stem cell differentiation. Wishbone, an algorithm for positioning single cells along differentiation trajectories with high resolution, has been shown to accurately recover the bifurcation point of T cell development using mass cytometry data [37]. Thus, we employed this computational analysis to identify the trajectory of signaling programs in HSC differentiation using our data from mice. LSK cells from normal mice were first analyzed and visualized using tSNE. Based on the markers’ expression, a single LT-HSC cell with a CD34^−^Flk2^−^CD48^−^CD150^+^ phenotype on the tSNE map was selected as a start point and a trajectory was subsequently established using Wishbone (Fig. 2b). We applied Wishbone to LSK cells from four independent mice and observed similar trajectory behaviors on the HSC markers’ expression crossing all samples (Fig. 2c), confirming the robustness of this analysis. Next, we deeply analyzed the trajectory of signaling proteins in one representative mouse. The expression dynamics of four key markers (Fig. 2d) and 12 signaling proteins along with the trajectory (Fig. 2e, and Additional file 1: Figure S1a) were analyzed, showing a diversity of signaling changes at different differentiating stages. From the trajectory, we were able to clearly observe the signaling transition from LT-HSC to LMPP through MPP (Fig. 2f) and the specific signaling programs in these transitions may regulate HSC self-renew and differentiation. According to the surface markers’ expression, the trajectory was divided into LT-HSC, MPP, and LMPP cell populations (Fig. 2f). Three stages showing distinct levels of β-catenin, pERK1/2, and IκBα in LT-HSCs and diverse levels of pSTAT3, pERK1/2, and IκBα in 2 MPP stages were observed (Fig. 2f). In addition, a clear gap of the signaling features was observed between MPP and LMPP stages, showing distinct signaling programs between them. In the LMPP, several parts of the trajectory showed diverse patterns of high signaling intensity (Fig. 2f), indicating a heterogeneity of pathway activation in these cells. Moreover, in the other individual mice, several clear stages could also be observed according to the abundances of signaling proteins and surface markers (Fig. 2f, and Additional file 1: S1b), highlighting the cellular heterogeneity of signaling programs in HSPCs.

### Signaling signatures during HSPC differentiation

**Fig. 3:**
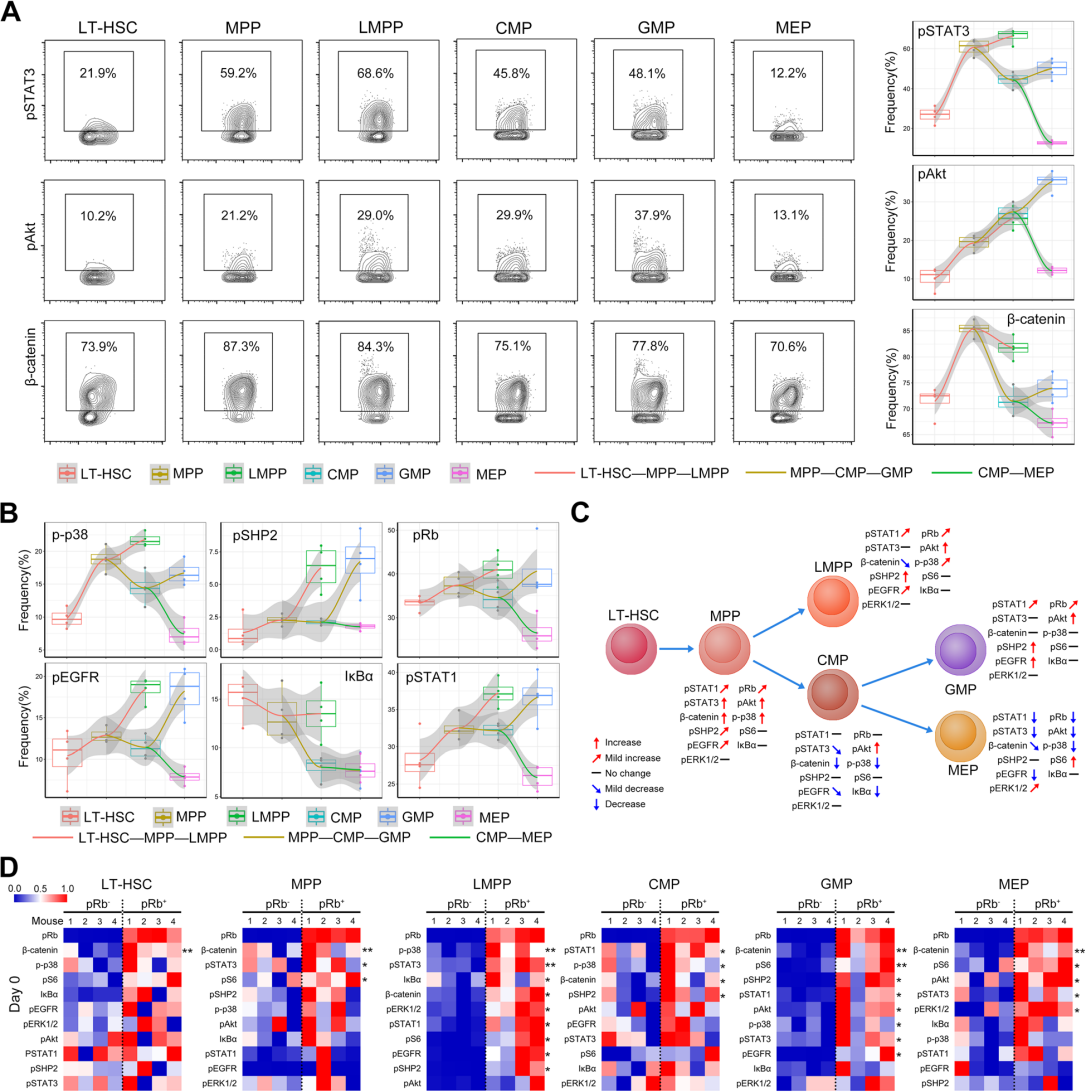
Signaling programs in HSPC subsets. **A** Representative contour plots for pSTAT3, pAkt, and β-catenin in different HSPC subsets. Combined charts of dot plots, box plots, and fitting curves showing the frequency changes of the indicated protein positive cells in different LK cell subsets in mice. Fitting curves in different colors indicate the changes of different differentiation routes. **B** Combined charts of dot plots, box plots, and fitting curves showing the frequency changes of p-p38, pSHP2, pRb, pEGFR, IκBα, and pSTAT1 positive cells in different LK cell subsets in mice. **C** Schematic diagram showing the signaling protein changes during HSC differentiation. **D** Heatmaps showing the normalized mean expression of the indicated signaling proteins in pRb^−^ and pRb^+^ HSPC subsets from mice. *p* values were calculated with paired Student’s *t* test. *n* = 4 mice in each group. *p < 0.05, **p < 0.01

We next analyzed the transitions of the general signaling status in HSPC subsets by showing the changes in the proportion of activated cells. With the analyses of frequency changes in HSPC subsets, plenty of regular signaling changes in all mice were demonstrated during the differentiation from LT-HSC to GMP or MEP (Fig. 3a, b). Specifically, elevated pAkt, p-p38, pSTAT3, and β-catenin levels in MPP compared to LT-HSC; distinct changes in the levels of pSHP2, pEGFR, p-p38, pSTAT3, IκBα, and β-catenin in between LMPP and GMP differentiation from MPP; and changes of pSHP2, pEGFR, pRb, pAkt, pSTAT1, p-p38, pSTAT3, and pS6 in between GMP and MEP differentiation from CMP were clearly observed (Fig. 3a, b, and Additional file 1: Figure S2), suggesting the potential role of these pathways in distinct differentiation routes (Fig. 3c).

Quiescent HSCs are in a slowly dividing state, while, by contrast, progenitor cells display rapid cycling. Upon stimulation or injury, HSCs can also rapidly enter the cell cycle and thus contribute to hematopoiesis [38, 39]. An increasing amount of studies have pointed to an important role for cell cycle signaling pathways in the control of HSC fate [40, 41]. The phosphorylation of Rb positively modulates the G_1_-S phase transition that decides cell cycle entry [42]. Since pRb is able to reflect the cell dividing state, we used it to define the cycling HSPC. Compared to quiescent (pRb^−^) cells, cycling LT-HSC expressed higher level of β-catenin, cycling MPP expressed more β-catenin, pSTAT3, and pS6. Cycling LMPP increasingly expressed most of the detected signaling proteins except pAkt. Cycling CMP expressed more pSTAT1, p-p38, β-catenin, and pSHP2. Cycling GMP highly expressed most of the detected signaling proteins except IκBα and pERK1/2. Cycling MEP expressed more β-catenin, pS6, pAkt, pSTAT3, and pERK1/2 (Fig. 3d). These differences support the existence of specific signaling signatures in cycling HSPC subsets.

### Intracellular signaling dynamic during hematopoietic reconstruction

**Fig. 4:**
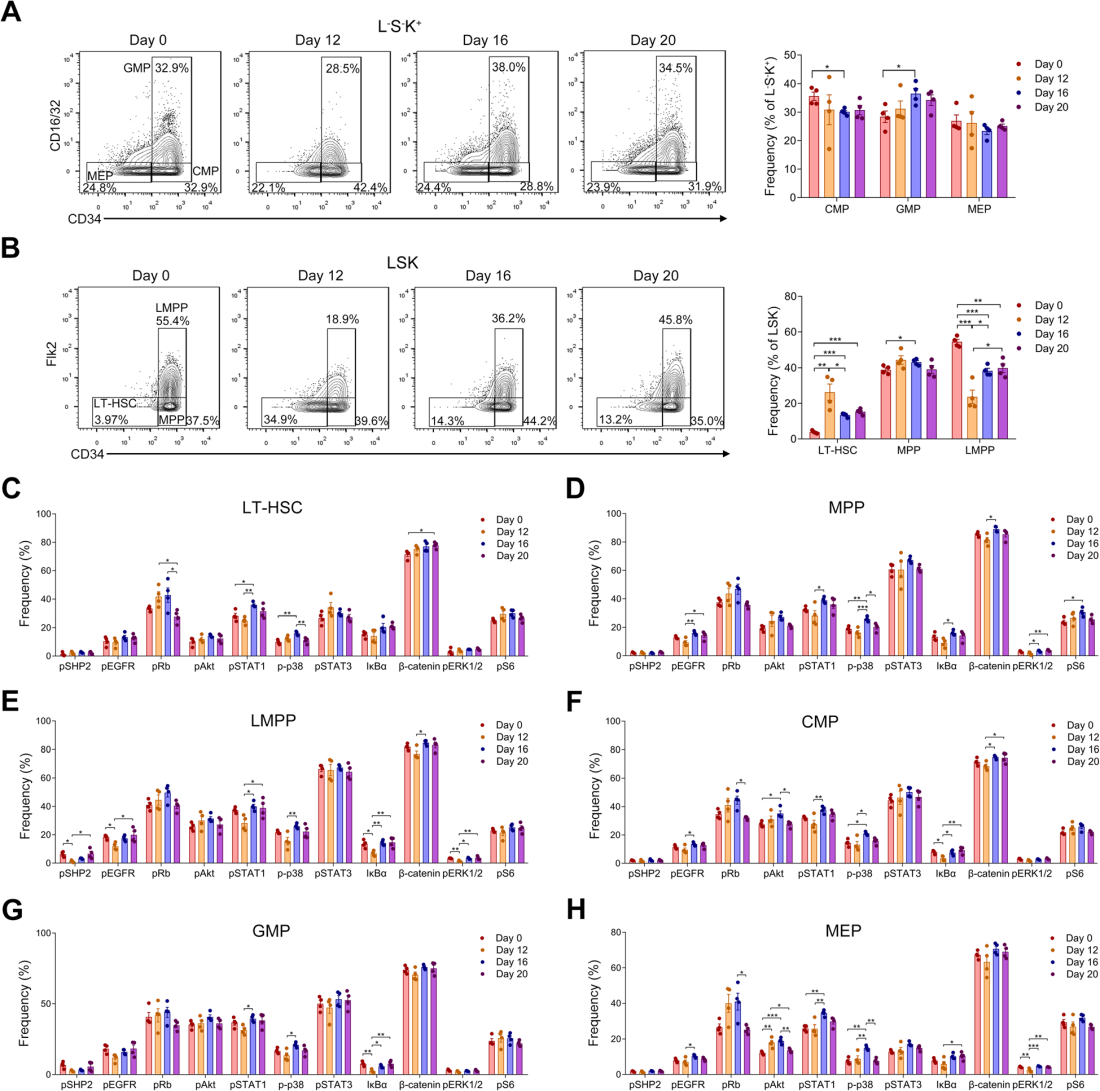
Signaling changes in HSPC subsets during hematopoietic recovery. Representative contour plots of **A** L^−^S^−^K^+^ and **B** LSK subsets after different days of 5-FU treatment (*n* = 4 mice in each group). Bar plots showing the frequencies of **A** CMP, GMP, MEP and **B** LT-HSC, MPP, and LMPP in the indicated cell populations after different days of 5-FU treatment. *p* values were calculated with Student’s *t* test. **C–H** Bar plots showing the frequencies of the indicated protein positive cells in **C** LT-HSC, **D** MPP, **E** LMPP, **F** CMP, **G** GMP, and **H** MEP cells from mice after different days of 5-FU treatment. *p* values were calculated with a one-way ANOVA followed by Tukey’s multiple comparisons test. *n* = 4 mice in each group. *p < 0.05, **p < 0.01, ***p < 0.001

5-Fluorouracil (5-FU) is a myelosuppressive drug widely used to study hematopoiesis. 5-FU-induced death of proliferating cells evokes the proliferation and differentiation of quiescent HSCs and thus serves as a valuable method to deeply investigate the processes of hematopoiesis [43]. Previous reports have shown that the expansion of HSPCs occurs from day 8 through day 20 after 5-FU treatment for increased hematopoietic demands [44, 45] and thus this period is considered to be critical for HSC regeneration and hematopoietic recovery. Therefore, we also treated mice with 5-FU for these periods to monitor the signaling changes during hematopoietic reconstitution. We analyzed and compared the signaling profiles in HSPC from mice experiencing recovery after 5-FU-induced hematopoietic injury. Our results show changes in HSPC subsets (Fig. 4a, b), including the significant increased proportion of LT-HSC in the LSK fraction at early recovery stage, which are consistent with previous reports [44, 45] and thus confirm that these mice are experiencing hematopoietic reconstruction.

To uncover the possible connection of different pathways in HSPC subsets during hematopoietic reconstruction, we evaluated the correlations between signaling protein levels. Several strong correlations (|*r*| > 0.4) between the expressions of surface markers or signaling proteins were observed and changed in mice treated with 5-FU for different days (Additional file 1: Figure S3a). In LT-HSC, MPP, CMP, and MEP subsets, only few strong correlationships between the expressions of detected proteins were discovered (Additional file 1: Figure S3b). By contrast, a number of strong correlations were found in GMP and LMPP cells (Additional file 1: Figure S3c and S3d), indicating that more activated signaling programs in these two progenitor subsets.

Frequencies of the signaling protein-expressing cells in each HSPC subset from mice at different stages of hematopoietic recovery were next quantified. In different subsets, distinct changes were revealed (Fig. 4c–h). In LT-HSC, pRb-expressing cells were elevated at the early stage of hematopoietic recovery and then decreased at day 20 after myelosuppression. During the reconstruction of hematopoietic system, the levels of pSTAT1, p-p38, and β-catenin were also regulated in LT-HSCs (Fig. 4c). In the other HSPC subsets, multiple signaling proteins were significantly regulated during hematopoietic recovery and the distinct regulatory patterns among these HSPC subsets were revealed (Fig. 4d–h). Of note, in HSPC subsets, the levels of different signaling proteins were always heterogeneous, suggesting that only a part of the cells are active. The frequency changes in several signaling proteins after 5-FU-induced hematopoietic activation were statistically significant, but these changes were always moderate, and this may be due to the fact that the regulation of hematopoietic restoration is a slow and gradual process.

### Identification of LSK subsets mainly responsible for hematopoietic reconstruction

**Fig. 5:**
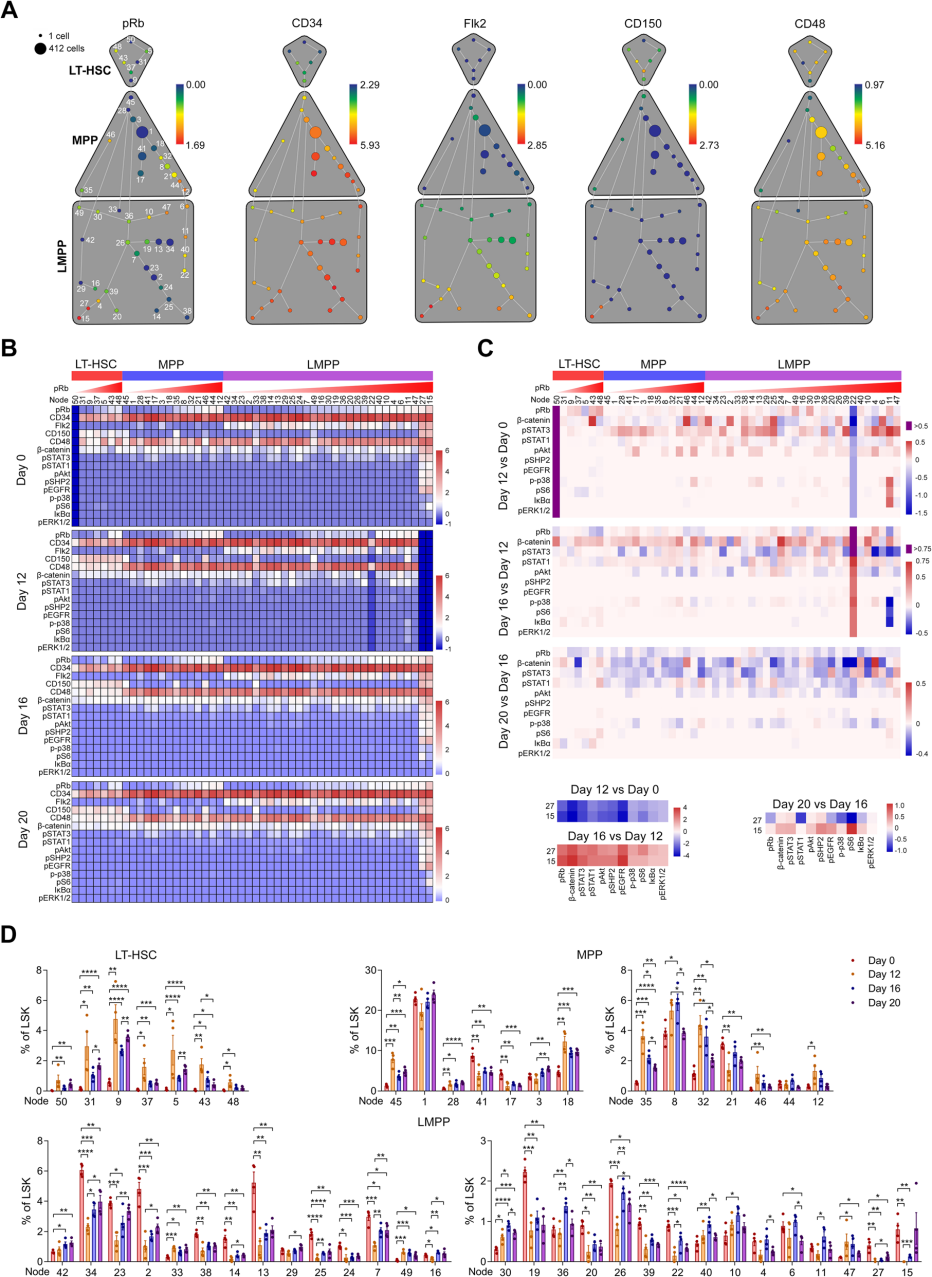
Characterization and changes of LSK cell clusters during hematopoietic recovery. **A** SPADE tree describing 50 minor LSK cell clusters of one representative mouse colored by the median expression of the indicated markers. LSK subsets are gated with a grey color. **B** Heatmaps showing the normalized median expression of the indicated markers in 50 minor LSK cell clusters in mice after different days of 5-FU treatment (*n* = 4 mice in each group). **C** Heatmaps displaying the differences in proteins’ expression levels in between LSK cells clusters in mice after the indicated days of 5-FU treatment. **D** Bar plots showing the frequency changes of LSK cell clusters (nodes) in mice after different days of 5-FU treatment. *n* = 4 mice in each group. *p < 0.05, **p < 0.01, ***p < 0.001, ****p < 0.0001. *p* values were calculated with Student’s *t* test

To systematically assess the signaling and subpopulation transitions during hematopoietic reconstruction, we introduced SPADE analysis [46] to enable cellular hierarchy inference among subsets of similar cells and distinguish the phenotypes of the most changed and activated minor LSK clusters. Fifty clusters containing different counts of cells (Additional file 2: Table S2) were derived from LSK cells and classified into LT-HSC (7 clusters), MPP (14 clusters), and LMPP (29 clusters) populations (Fig. 5a, and Additional file 1: Figure S4) after surface marker-based phenotyping. Using cytoclusterR, heterogeneity of signaling signatures across 50 LSK cell clusters from mice after 0, 12, 16, or 20 days of 5-FU treatment was clearly presented on heatmaps (Fig. 5b). Additionally, the differences of the median expression of signaling protein and markers in each cluster from mice at different recovery stages were summarized and multiple changes of all the signaling proteins in different clusters were observed over time (Fig. 5c). Specifically, LT-HSCs in node 50 with a CD34^−^Flk2^−^CD150^−^CD48^int^ phenotype, localized at the starting point of the cellular hierarchy tree, was virtually non-existent in mice without treatment (day 0), whereas it expanded greatly at day 12 post 5-FU treatment with moderate expressions of pRb and β-catenin, implicating that these cells may represent the self-renewing stem cells. pRb and β-catenin were changed in cluster 43 and 48 at day 12 post treatment. Nodes 15 and 27 with multiple signaling activations in normal mice were absent at day 12 post treatment, and this may be due to the fact that these cells were highly proliferative and thus preferably suppressed by 5-FU. At day 16 post myelosuppression, these cells were present since they were differentiated again from their progenitor cells (Fig. 5c). Furthermore, proportions of 18 clusters, including all 7 LT-HSC, 7 MPP (cluster 45, 28, 18, 35, 32, 46, and 12), and 4 LMPP clusters (cluster 33, 49, 30, and 47), were increased at the early stage (day 12) (Fig. 5d), suggesting that these cells may be the most activated stem or precursor cells responsible for the lineage differentiation and hematopoietic reconstruction.

### Signaling changes in BM lineages along with hematopoiesis remodeling

**Fig. 6:**
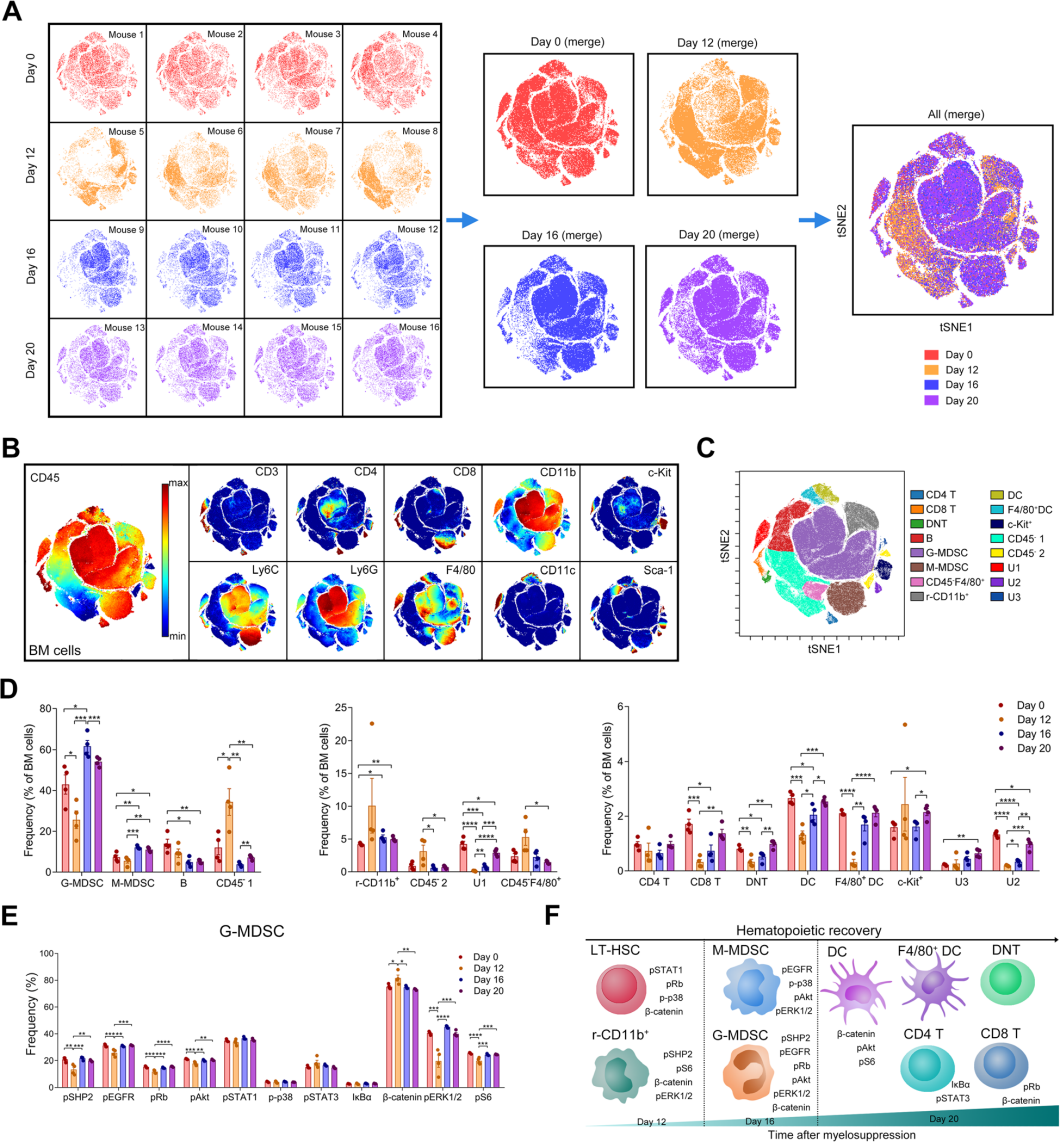
Signal proteins changes in BM subsets during hematopoietic recovery. **A** viSNE map displaying BM cells of all the mice (15,400 cells from each mouse). **B** viSNE maps colored by the normalized expression of the indicated markers. **C** viSNE map colored by 16 cell populations after clustering. **D** Bar plots showing the frequencies of BM cell subsets after different days of 5-FU treatment. *p* values were calculated with Student’s *t* test. **E** Bar plots showing the frequencies of the indicated protein positive cells in G-MDSCs from mice after different days of 5-FU treatment. *p* values were calculated with a one-way ANOVA followed by Tukey’s multiple comparisons test. **F** Schematic diagram showing the recovery process of BM cell subsets and the changed signaling proteins in each cell population after myelosuppression. *n* = 4 mice in each group. *p < 0.05, **p < 0.01, ***p < 0.001

We generated a single-cell viSNE map to visualize high-dimensional data in two dimensions [47] using BM cell data from all mice and evaluated the changes of BM lineage cells after myelosuppression (Fig. 6a). According to the phenotypes of immune cell populations (Additional file 1: Figure S5a) and to the expression of lineage markers in cells on the viSNE map (Fig. 6b), 16 cell populations, including several undefined or CD45- cell subsets, were clustered after manual gating (Fig. 6c). Based on the heatmap (Additional file 1: Figure S5b), we observed that 12 markers expressed in each population were identical to their phenotype. From the viSNE maps of mice at different recovery stages, cell distribution changes were clearly observed (Additional file 1: Figure S5c). The frequencies of each cell population were summarized and compared (Additional file 1: Figure S5d). The average percentages of CD45^−^F4/80^+^, CD45^−^, and the rest of CD11b^+^ cells (r-CD11b^+^) at 12 days after myelosuppression were higher than those at day 0, suggesting that these cells may be less sensitive to 5-FU or become quickly differentiated from activated HSC. Sixteen days post treatment, proportions of granulocytic myeloid-derived suppressor cells (G-MDSC) and monocytic MDSC (M-MDSC) were recovered. The percentages of lymphoid lineages, such as T cell subsets, DC, and F4/80^+^DC were returned to the level at day 0 (Fig. 6d).

Next, we analyzed signaling protein levels in lineage cell subsets. Quantification of the frequency of all the signaling protein positive cells revealed a diversity of signaling status in different cell subsets during hematopoietic recovery (Fig. 6e and Additional file 1: Figure S6a-g). After myelosuppression, G-MDSC displayed massive changes of signaling protein levels at day 12 post treatment (Fig. 6e). Distinct signaling changes in lineage subsets during hematopoiesis remodeling implicate that specific signaling pathways are involved in certain lineage differentiations (Fig. 6f).

### Signaling programs involved in lineage differentiation

**Fig. 7:**
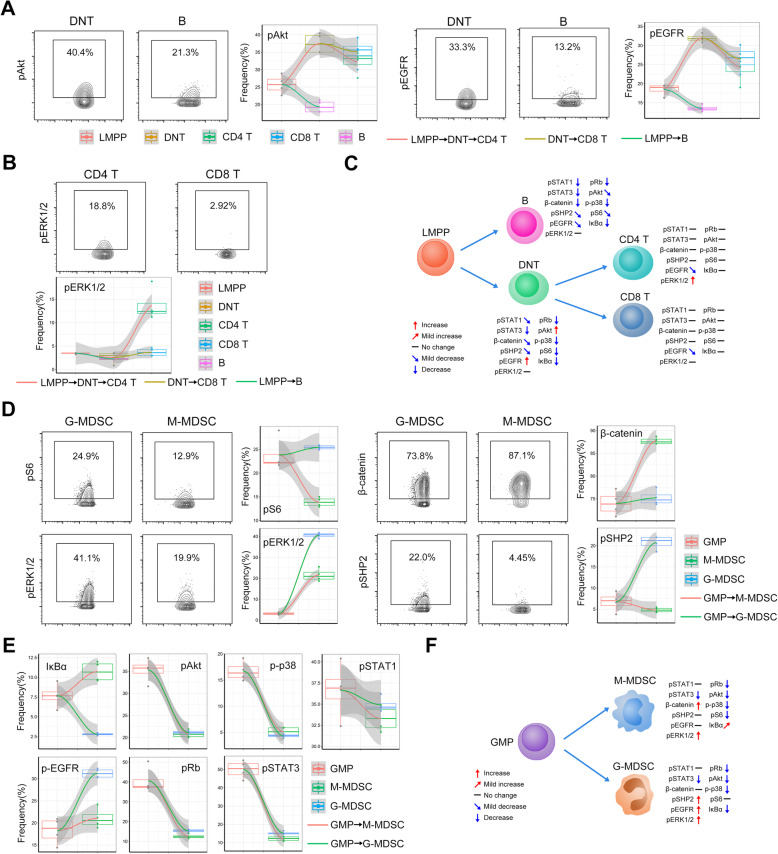
Signaling changes in lineage subsets during hematopoietic recovery. **A** Representative contour plots for pAkt and pEGFR and in DNT and B cells. Combined charts of dot plots, box plots, and fitting curves showing the frequency changes of the indicated protein positive cells in different lineage cell subsets in mice. Fitting curves in different colors indicate the changes of different differentiation routes from LMPP to T cell subsets or B cells. **B** Representative contour plots for pERK1/2 in CD4 and CD8 T cells. Combined charts of dot plots, box plots, and fitting curves showing the frequency changes of pERK1/2-positive cells in different lineage cell subsets in mice. **C** Schematic diagram showing the signaling protein changes during lymphocyte differentiation. **D** Representative contour plots for pS6, pERK1/2, β-catenin, and pSHP2 and in G-MDSC and M-MDSC. **D, E** Combined charts of dot plots, box plots, and fitting curves showing the frequency changes of the indicated protein positive cells in different lineage cell subsets in mice. Fitting curves in different colors indicate the changes of different differentiation routes from GMP to G-MDSC or M-MDSC. **F** Schematic diagram showing the signaling protein changes during myeloid cell differentiation. *n* = 4 mice in each group

According to these frequencies, we established multiple trajectories describing the changes of signaling protein expression in differentiation from precursor to lineage cells under normal conditions or at different recovery stages of hematopoiesis. The differences in the expression of pAkt, pEGFR, pS6, and β-catenin were the greatest in between the differentiations from LMPP to T and B lymphocytes (Fig. 7a and Additional file 1: Figure S7a). A sharp difference in pERK1/2 expression was found in between CD4 and CD8 T cells (Fig. 7b and c). pS6, pERK1/2, β-catenin, pSHP2, IκBα, and pEGFR differed greatly between G-MDSC and M-MDSC differentiation from GMP (Fig. 7d, e, and Additional file 1: Figure S7b), emphasizing the role of these signaling programs in lineage differentiation.

## Discussion

The revolution of single-cell analysis is substantially transforming our understanding of hematopoietic hierarchy and behavior, and significantly promoting discovery more complete analysis of hematopoiesis. Here, we took advantage of an advanced single-cell profiling technology to capture a signaling dynamic across HSPC differentiation and lineage cell development in hematopoiesis and hematopoietic recovery at the protein level. Signaling changes in the multiple lineage cell differentiations from HSC were comprehensively analyzed, such as Jak/Stat, MAPK/P38, and NF-κB signaling in between LMPP and CMP differentiation, Jak/Stat, Wnt/β-catenin, MAPK/ERK/p38, and PI3K/Akt signaling in between GMP and MEP differentiation (Fig. 3c), Jak/Stat, MAPK/ERK, and PI3K/Akt signaling in between B and T differentiation, MAPK/ERK signaling in between CD4 and CD8 T development, and Wnt/β-catenin, mTOR, and NF-κB signaling in between M- and G-MDSC differentiation (Fig. 7c, f). In addition, major signaling pathways involved in hematopoietic cell recovery were also demonstrated (Fig. 6f). Importantly, we show for the first time a reconstruction of the dynamics of signaling changes during hematopoietic cell fate diversification. Our mass cytometry data extends our knowledge about hematopoiesis and complements the deficiency in transcriptomic datasets by providing the hematopoiesis research community an intracellular signaling atlas at the single-cell resolution. The discovery of these patterns and changes will also facilitate the development of strategies for controlling HSC in vitro expansion and directional differentiation by programing specific signaling activation.

Previous studies have evaluated the signaling differences in human hematopoietic cells and across HSC differentiation using mass cytometry. Bendall et al. [31] have used mass cytometry to examine the levels of 18 intracellular functional markers in a variety of human hematologic cell types after ex vivo stimulation or inhibition. After comprehensive analysis, they provided a superimposable map of signaling responses in combination with growth factor stimulation or drug inhibition. Another study has investigated the signaling programs controlling human HSC survival and proliferation in vitro using mass cytometry [48]. They measured active signaling intermediates in single CD34^+^ cord blood cells before and after stimulation with multiple growth factors, and thus provided the molecular mechanisms by which growth factors modulate the survival and proliferation of human HSCs. Additionally, they found that human CD34^+^ cord blood cells are heterogeneous in signaling activities and their in vitro datasets revealed a multiplicity of lineage restriction trajectories [18]. These studies have systematically analyzed the intracellular signaling changes in human hematopoietic stem and lineage cells in response to the stimulation of growth factors and thus confirmed close relationships between growth factor-induced signaling activation and HSC proliferation, survival, and differentiation. Here, we took advantage of an animal model to assess the signaling programs in primary hematopoietic cells directly obtained from a homeostatic in vivo environment or undergoing recovery. Without extra stimulation and in vitro culture, the in vivo signaling statues were maximally retained and thus these data can better reflect the actual changes in vivo. Further, with the development of alignment algorithms for HSC analyzing, hematopoiesis is increasingly regarded as a spectrum of cell types. Therefore, the binning into discrete states as visualized and interpreted in our results relies on the suspension of the fact that cells are exhibiting gradations of multiple signaling pathway activations.

After quantification of the signaling status in HSPC subsets, regular changes of multiple signaling proteins, representing Jak/Stat, PI3K/Akt, MAPK, NF-κB, mTOR, and Wnt signaling pathways and cell replication activation, appeared in different HSC differentiation routes. Wnt/β-catenin signaling has been suggested to play a pivotal role in the regulation of HSC as early as two decades ago when Wnt was shown to stimulate the survival and proliferation of hematopoietic progenitors [49]. Cumulatively, more and more studies validated the regulatory role of Wnt/β-catenin signaling in hematopoiesis by maintaining quiescence and balance in HSC proliferation [50]. Additionally, Wnt/β-catenin signaling is differentially regulated in HSPC subsets and is able to dose-dependently modulate HSCs, myeloid precursors, and T lymphoid precursors during hematopoiesis [51, 52]. Mild Wnt signaling promotes HSC function, whereas high levels are detrimental. Intermediate Wnt signaling enhances myeloid and T cell development, but B cell development is irrelative with Wnt activities [52]. Intriguingly, our single-cell data also demonstrate a distinct β-catenin expression during HSC differentiation and reveal various transitions of β-catenin signaling in myeloid and lymphoid precursor differentiation from MPP, in B and T cell differentiation from LMPP, as well as in G-and M-MDSC differentiation. These discoveries reinforce the role of Wnt/β-catenin signaling in hematopoiesis and further provide invaluable clues for identifying the involvement of different levels of Wnt signaling activation in HSC fate decision.

Jak/Stat, PI3K/Akt, and MAPK signaling extensively regulate cell proliferation, differentiation, quiescence, cycle, migration, and apoptosis and thus are involved in a wide variety of critical cellular processes, such as hematopoiesis, immune development and response, metabolism, and tumor formation [53–55]. The activation of these pathways is also regulated in hematopoiesis, especially in thrombopoietin (TPO)-mediated megakaryopoiesis [50, 56]. Upon the binding of TPO to its receptor c-Mpl which is mainly expressed on HSC, the downstream cascade STATs, PI3K/Akt, and MAPK/ERK1/2 are activated and therefore maintain steady-state and cell cycle progression of HSC [57–59]. Our data show a relatively low level of STATs, Akt, and p38 activation in LT-HSC and diverse transitions of their activities in the differentiation into different progenitor and lineage cells, supporting the participation of these pathways in hematopoiesis. Among the pathways we detected, only distinct ERK1/2 activation was clearly observed between CD4 and CD8 T cells, indicating its potential role in T cell development. Indeed, ERK cascade is one of the first pathways identified as crucial for T cell positive selection [60] and T cell development and activation is severely impaired in ERK1^−/−^ mice [61], testifying to the robustness of our single-cell data regarding the distinctive character of ERK1/2 activation between T cell subsets.

NF-κB signaling represents a pivotal regulator and mediator of innate and adaptive immune functions and inflammatory responses. IκBα negatively regulates its activation by binding to NF-κB protein dimmers and interfering with their nuclear localization [62]. Basal activity of NF-κB signaling is required for the homeostasis of HSCs [63] and experimental induction of NF-κB signaling results in an activation and expansion of HSPC as well as enhanced myeloid differentiation [64–66]. These results are consistent with our data showing a significantly lower IκBα expression in CMP compared to LMPP and their progenitor MPP, and further emphasize the important and positive role for NF-κB activation in myeloid differentiation. Depletion of IκBα-induced constitutive NF-κB activation can lead to granulopoiesis in mice [67]. Our date showed a significantly lower level of IκBα in G-MDSC, precursors of granulocytes, compared to their differentiation competitor M-MDSC and their progenitor GMP, indicating that the activation of NF-κB signaling may determine the granulocytic differentiation. Our results demonstrating the transition patterns of signaling among HSPC and lineage subsets are in line with the aforementioned experimental results, therefore validating the reliability of signal-cell data and underlining the supportive role of signaling profiling in hematopoiesis research, especially in HSC fate decision studies.

Although surface marker-based identification of HSPC subsets has been widely used in HSC research, increasing single-cell studies have confirmed the heterogeneity within HSPC subsets and revealed that some of them consist of heterogeneous mixture of other cell types [23, 68]. Here, although we found diverse patterns of signaling in HSPC subsets, several underlying limitations are raised. First, it remains unclear whether these signaling diversities are caused by the heterogeneity of cell types. Second, the meaning of the signaling patterns in HSPC differentiation cannot be ultimately validated without any function experiments. As signaling regulation in HSC differentiation is complex and delicate, extensive experiments are required to confirm our findings. Furthermore, comprehensive single-cell analysis involving transcriptomics and proteomics, as well as functional validation, is warranted to verify the heterogeneity in HSPC subsets during HSC differentiation and to identify robust markers to phenotype those HSPC subsets.

## Conclusion

Overall, our work has profiled the signaling landscape and transitions across hematopoietic cell subsets during hematopoiesis and hematopoietic recovery. Moreover, it mapped trajectories, whereby the intracellular signaling activation is accurately described in hematopoiesis, and multiple signaling targets that may serve as critical mediators in HSC lineage branching are provided. These comprehensive single-cell data covering almost all of the BM hematopoietic cell types will largely facilitate the identification of detailed mechanisms of HSC self-renewal and differentiation as well as the principles underlying stem cell fate decisions. Our results not only validate previous discoveries but also provide substantial new evidence and clues to renew and improve our understanding of hematopoietic hierarchy.

## Methods

### Mice

Six-to-eight-week-old female C57BL/6 mice were purchased from Guangdong Medical Laboratory Animal Center and all these mice were housed and fed in specific-pathogen-free animal care facilities at Guangzhou Medical University. All procedures were approved by the Ethical Committee for Animal Experiments of the Guangzhou Medical University.

### 5-FU treatment

A single dose of 5-FU (150 mg/kg, Sigma) in Dulbecco’s phosphate buffered saline (DPBS) was intraperitoneally administered to mice after 1 week acclimatization in the animal care facilities at Guangzhou Medical University.

### BM cell isolation

Four mice were sacrificed at day 0 (without injection), 12, 16, and 20 after 5-FU treatment, and the BM cells were immediately isolated from tibias and femurs. The freshly isolated BM cells were filtered with a 70-μm cell strainer and immediately fixed with Fix I buffer (Fluidigm) for 5 min at RT. Fixed cells were washed twice with cell staining buffer (CSB, DPBS containing 0.5% bovine serum albumin and 0.02% sodium azide), and red blood cells were removed using ammonium-chloride-potassium lysis buffer. Cells were resuspended in CSB containing 10% DMSO and stored at − 80 °C.

### Magnetic-activated cell sorting (MACS) for HSPC enrichment

Most of the fixed cells were washed twice with CSB and stained with biotin anti-mouse Lineage Panel containing biotinylated antibodies against mouse TER-119, CD11b, Ly-6G/Ly-6C (Gr-1), CD3e, and B220 for 15 min at RT. These cells were washed twice with MACS buffer (DPBS supplemented with 0.5% FBS and 2 mM EDTA) and incubated with streptavidin microbeads (Miltenyi Biotec, Bergisch Gladbach, Germany) for 15 min at 4 °C. Lineage^−^ cells were separated using LS Columns (Miltenyi Biotec) and a MACS separator (Miltenyi Biotec).

### Barcoding

All lineage^−^ or BM cells isolated from each mouse were barcoded first and then stained, processed, and acquired as one multiplexed sample to eliminate sample-specific staining variation. 0.5 × 10^6^ fixed lineage^−^ or BM cells from each mouse were washed 3 times with CSB and washed twice with 1× Barcode Perm Buffer (Fluidigm). These samples were then separately barcoded using a 20-Plex Pd Barcoding Kit (Fluidigm) according to the manufacturer’s instruction. All samples were separately washed 3 times with CSB and lineage^−^ and BM samples were combined together separately into two tubes for antibody staining.

### Antibody staining

Barcoded BM samples were incubated with purified anti-CD16/32 (Biolegend) for 10 min at RT to lower nonspecific antibody binding. Anti-mouse CD3e-FITC, Ly6G-APC, and Ly6C-biotin were added to the BM sample after Fc-blocking. Barcoded lineage^−^ samples were incubated with anti-CD16/32-144Nd for 10 min at RT, and then anti-mouse CD34-FITC and anti-mouse Flk2-APC were added to the sample. These samples were stained with these antibodies for 30 min at RT and washed twice with CSB. Lineage^−^ samples were stained with metal isotope-tagged antibodies against 4 surface markers and 3 metal-labeled secondary antibody against biotin, FITC, or APC (Table S1, HSPC panel) for 30 min at RT. BM samples were incubated with metal isotope-tagged antibodies against 9 surface markers and 3 metal-labeled secondary antibody against biotin, FITC, or APC (Table S1, BM panel) for 30 min at RT. Samples were then washed 3 times with CSB and resuspended in 100 μl CSB and placed on ice for 10 min. One milliliter 4 °C methanol was added to each sample and incubated for 15 min on ice. Samples were washed 3 times with CSB and incubated with metal isotope-tagged antibodies against 12 intracellular proteins (Table S1, Intracellular protein panel) for 30 min at RT. These cells were washed 3 times with CSB and resuspended in 1 ml Fix & Perm Buffer (Fluidigm) containing 125 nM Intercalator-Ir (Fluidigm) overnight at 4 °C.

### CyTOF Data Acquisition

Stained samples were washed twice with CSB and ultrapure water, respectively. Immediately prior to data acquisition, the cells were suspended in ultrapure water containing 15% EQ Four Element Calibration Beads (Fluidigm) and filtered through a 400 mesh cell strainer. All samples were acquired on a Helios mass cytometer (Fluidigm). CyTOF software 6.7 (Fluidigm) was used to normalize and debarcode the data.

### Mass cytometry data analyses

Debarcode files were analyzed using an online single-cell analyzer, Cytobank. All residual EQ beads, cell debris, and doublets, as well as cells with abnormal even length, were excluded from the events. Contour plots and frequencies of cell subsets was determined using Flowjo software (Flowjo, LLC). For setting a proper threshold for scoring positivity, the minimum and maximum proportions of phosphorylated proteins in populations of our data were obtained and then the gate was adjusted based on the population with no or a small number of positive cells. viSNE, SPADE, and heatmaps were implemented using Cytobank. Box plot, histogram, and heatmap of correlationship were generated using GraphPad. The SPADE nodes were implemented using the cytoClustR R package developed by Kordasti Lab from King’s College London. Pearson correlations analysis was performed using SPSS 20.0 software. Wishbone trajectory was completed using a python package for Wishbone algorithm. A *κ* value of 15, 250 waypoints, and diffusion components 1, 2, and 3 were used for Wishbone analysis. The charts that combine dot plots, box plots, and fitting curves were generated using ggplot2 with the frequencies of indicated cell population or expressions of indicated signaling protein.

### Statistical analyses

Shapiro-Wilk test was used to test whether the data is normally distributed. Student’s *t* test or paired *t* test was used to determine the statistical significance between 2 groups. One-way ANOVA followed by Tukey’s multiple comparisons test was used for comparing multiple groups. Correlation analyses between population frequencies were performed using Person’s correlation coefficient. Error bars represent mean ± standard error of mean (S.E.M.). *p* < 0.05 was regarded as statistically significant.

## Supplementary Information

**Additional file 1: Figure S1.** (A) Plots comparing the dynamics of the indicated proteins along the trajectory in LSK cells obtained from mouse 2 (*n* = 4950 LSK cells), 3 (*n* = 4106 LSK cells) and 4 (*n* = 2890 LSK cells). (B) Derivative plot showing the changes in expression of the indicated proteins along the trajectory in mouse 3 and 4. HSPC subsets and stages were divided using dotted lines. Arrows indicate the main signaling proteins in LT-HSC. Color bars indicate the normalized expression level. **Figure S2.** Combined charts of dot plots, box plots, and fitting curves showing the frequency changes of pS6 and pERK1/2 positive cells in different LK cell subsets in mice. **Figure S3.** (A) Bar plots showing the changes of Pearson correlation coefficients for relationships (r > 0.4) between the expressions of the indicated proteins in LSK cells from mice after different days of 5-FU treatment. (B) Bar plots showing the changes of Pearson correlation coefficients for relationships (r > 0.4) between the expressions of the indicated markers in LT-HSC, MPP, CMP, and MEP cells from mice after different days of 5-FU treatment. (C and D) Heatmap showing Pearson correlation coefficients for relationships (r > 0.4) between the expressions of the indicated markers in (C) LMPP and (D) GMP cells from mice after different days of 5-FU treatment. *n* = 4 mice in each group. **Figure S4.** SPADE trees describing 50 minor LSK cell clusters of one representative mouse from each group were colored by the median expression of the indicated markers. **Figure S5.** (A) Phenotypes for gating populations. (B) Heatmaps showing the normalized median expression of 12 markers in all cell populations. (C) viSNE map showing colored BM cell populations in representative mice from each group. (D) Frequencies of 16 cell populations in BM cells from each mouse. Cell types are indicated by color. *n* = 4 mice in each group. **Figure S6.** (A-G) Bar plots showing the frequencies of the indicated protein positive cells in 10 cell populations from mice after different days of 5-FU treatment. *n* = 4 mice in each group. **Figure S7.** (A) Combined charts of dot plots, box plots, and fitting curves showing the frequency changes of the indicated protein positive cells in different lineage cell subsets in mice. Fitting curves in different colors indicate the changes of different differentiation routes from LMPP to T cell subsets or B cells. (B) Representative contour plots for IκBα and pEGFR in G-MDSC and M-MDSC. Combined charts of dot plots, box plots, and fitting curves showing the frequency changes of IκBα and pEGFR positive cells in different lineage cell subsets in mice. Fitting curves in different colors indicate the changes of different differentiation routes from GMP to G-MDSC or M-MDSC. *n* = 4 mice in each group.

**Additional file 2: Table S1.** Mass cytometry antibody reagents. **Table S2.** Cell number in each SPADE node of each sample.

## Data Availability

All data generated or analyzed during this study are included in this published article, its supplementary information files, and publicly available repositories. All mass cytometry and original data are publicly available at Figshare: Wang, Jinheng (2021): Single-cell analysis at the protein level delineates intracellular signaling dynamic during hematopoiesis-mass cytometry +original data. figshare. Dataset. 10.6084/m9.figshare.16528836.v5.

## Abbreviations

HSPC: Hematopoietic stem and progenitor cell
5-FU: 5-Fluorouracil
BM: Bone marrow
CMP: Common myeloid progenitor
DNT: Double negative T
GMP: Granulocyte-macrophage progenitor
G-MDSC: Granulocytic myeloid-derived suppressor cell
LT-HSC: Long-term-hematopoietic stem cell
LMPP: Lymphoid-primed multipotent progenitor
MPP: Multipotent progenitor
MEP: Mgk/erythroid progenitor
M-MDSC: Monocytic myeloid-derived suppressor cell
TPO: Thrombopoietin
