# Supplementary figures and images for: Single-cell analysis at the protein level delineates intracellular signaling dynamic during hematopoiesis
Source: BMC Biol. 2021 Sep 9;19:201. doi: 10.1186/s12915-021-01138-6 (PMC8428103; doi:10.1186/s12915-021-01138-6)

Fig. S1

A

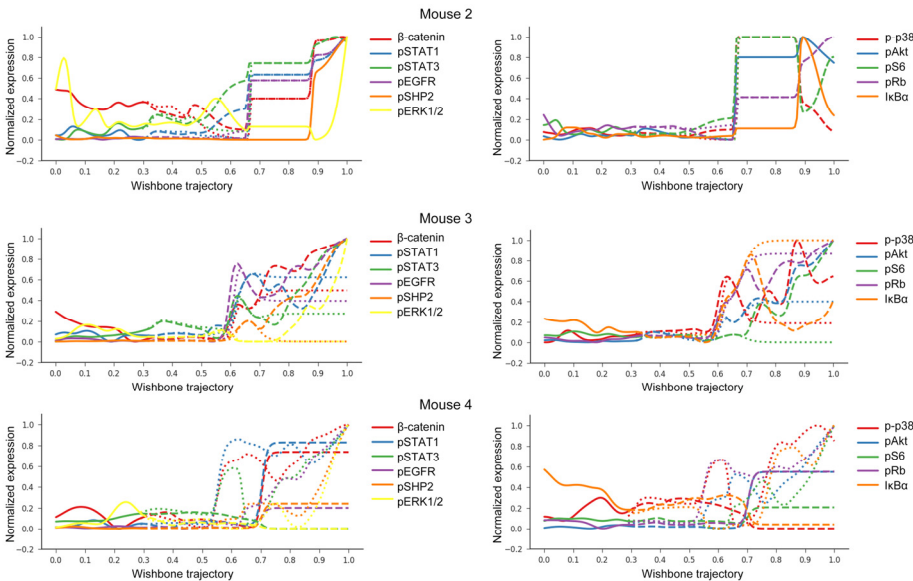

B

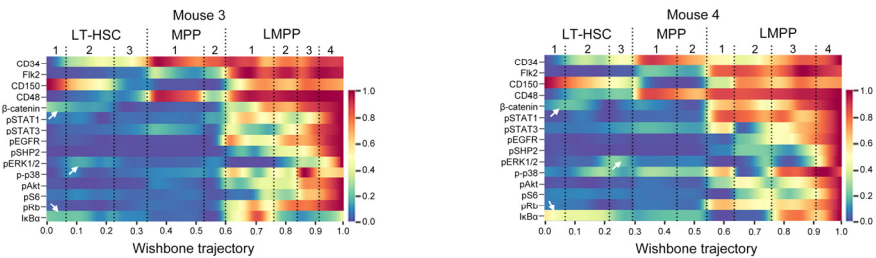

Fig. S2

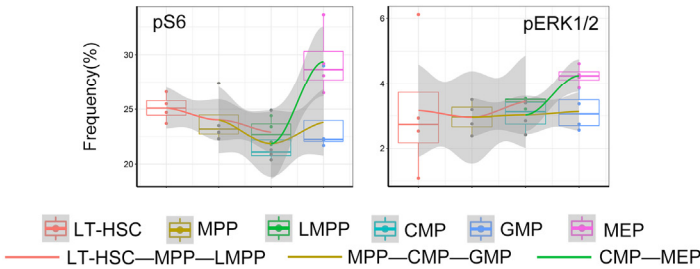

Fig. S3

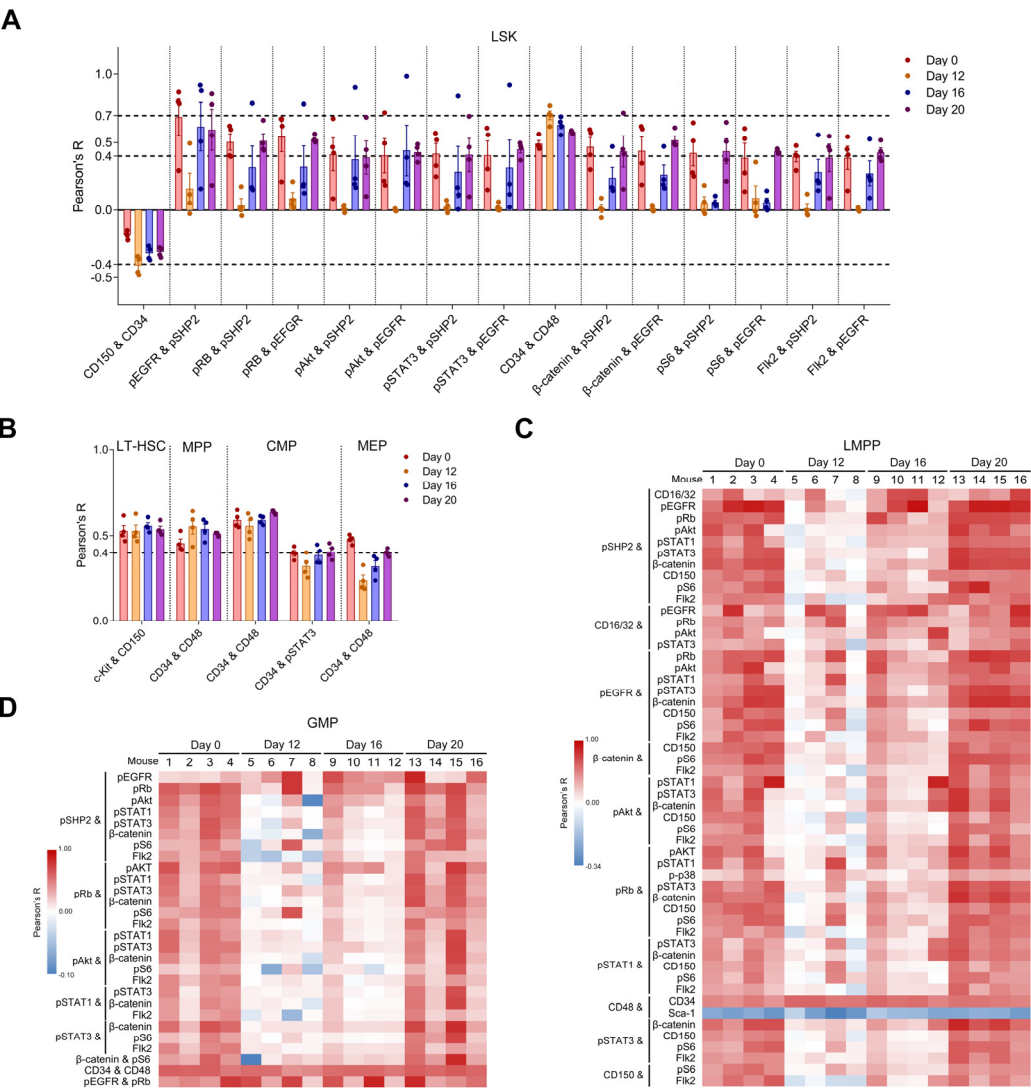

Fig. S4

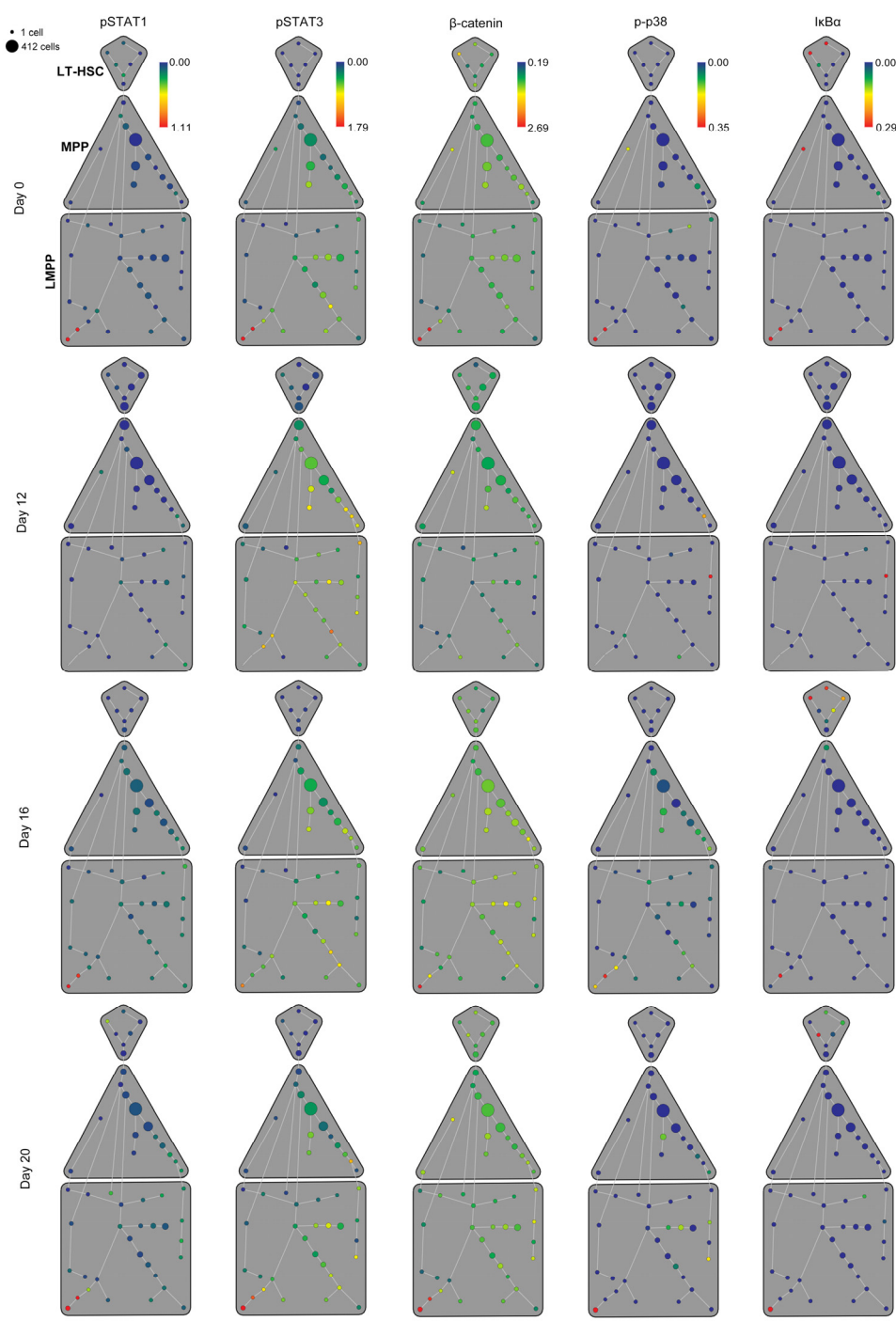

Fig. S5

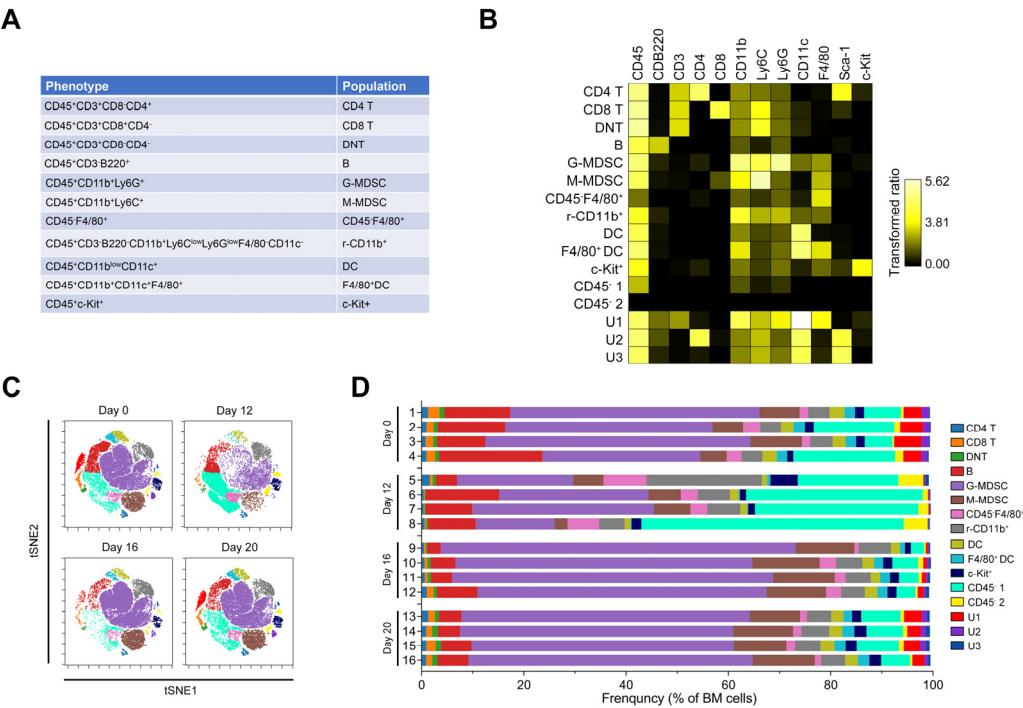

Fig. S6

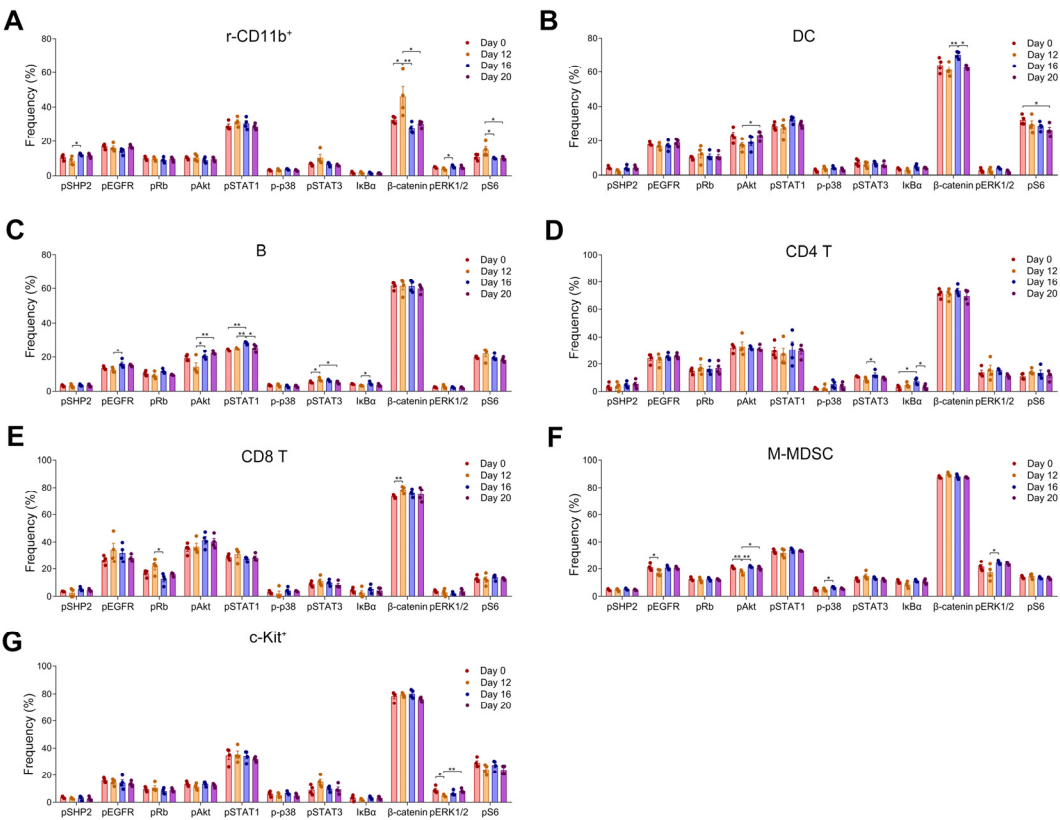

Fig. S7

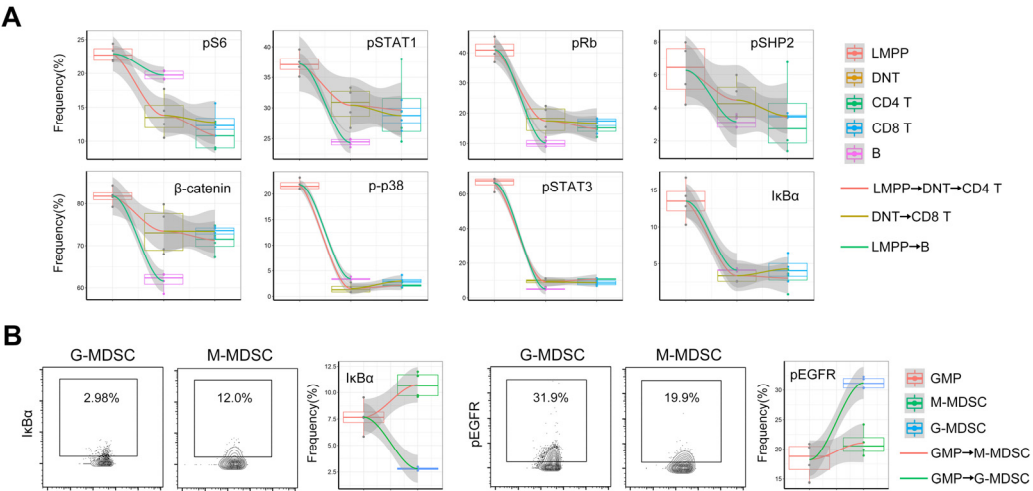

Supplement: Supplementary file 1 — Additional file 1: Figure S1. (A) Plots comparing the dynamics of the indicated proteins along the trajectory in LSK cells obtained from mouse 2 (n = 4950 LSK cells), 3 (n = 4106 LSK cells) and 4 (n = 2890 LSK cells). (B) Derivative plot showing the changes in expression of the indicated proteins along the trajectory in mouse 3 and 4. HSPC subsets and stages were divided using dotted lines. Arrows indicate the main signaling proteins in LT-HSC. Color bars indicate the normalized expression level. Figure S2. Combined charts of dot plots, box plots, and fitting curves showing the frequency changes of pS6 and pERK1/2 positive cells in different LK cell subsets in mice. Figure S3. (A) Bar plots showing the changes of Pearson correlation coefficients for relationships (r > 0.4) between the expressions of the indicated proteins in LSK cells from mice after different days of 5-FU treatment. (B) Bar plots showing the changes of Pearson correlation coefficients for relationships (r > 0.4) between the expressions of the indicated markers in LT-HSC, MPP, CMP, and MEP cells from mice after different days of 5-FU treatment. (C and D) Heatmap showing Pearson correlation coefficients for relationships (r > 0.4) between the expressions of the indicated markers in (C) LMPP and (D) GMP cells from mice after different days of 5-FU treatment. n = 4 mice in each group. Figure S4. SPADE trees describing 50 minor LSK cell clusters of one representative mouse from each group were colored by the median expression of the indicated markers. Figure S5. (A) Phenotypes for gating populations. (B) Heatmaps showing the normalized median expression of 12 markers in all cell populations. (C) viSNE map showing colored BM cell populations in representative mice from each group. (D) Frequencies of 16 cell populations in BM cells from each mouse. Cell types are indicated by color. n = 4 mice in each group. Figure S6. (A-G) Bar plots showing the frequencies of the indicated protein positive cells in 10 c [file 12915_2021_1138_MOESM1_ESM.pdf]
