# Supplementary material for: Single-cell analysis at the protein level delineates intracellular signaling dynamic during hematopoiesis
Source: BMC Biol. 2021 Sep 9;19:201. doi: 10.1186/s12915-021-01138-6 (PMC8428103; doi:10.1186/s12915-021-01138-6)
Supplement: Supplementary file 2 — Additional file 2: Table S1. Mass cytometry antibody reagents. Table S2. Cell number in each SPADE node of each sample. [file 12915_2021_1138_MOESM2_ESM.docx]

Table S1. Mass cytometry antibody reagents

| **Label** | **Target** | **Ab clone** | **Dilution** | **Catalog #** | **RRID** | **Source** |
| --- | --- | --- | --- | --- | --- | --- |
| **HSPC (lineage^-^) panel** | | | | | | |
| 173Yb | c-Kit | 2B8 | 1:100 | 3173004B | AB_2811230 | Fluidigm |
| 167Er | CD150 | TC15-12F12.2 | 1:50 | 3167004B | AB_2811231 | Fluidigm |
| 144Nd | CD16/32 | 93 | 1:100 | 3144009B | AB_2814674 | Fluidigm |
| 154Sm | CD48 | HM48-1 | 1:100 | 3154004B | AB_2811232 | Fluidigm |
| 169Tm | Sca-1 | D7 | 1:100 | 3169015B | AB_2811233 | Fluidigm |
| 170Er | Biotin | 1D4-C5 | 1:100 | 3170003B | AB_2811234 | Fluidigm |
| 160Gd | FITC | FIT-22 | 1:100 | 3160011B | AB_2811235 | Fluidigm |
| 176Yb | APC | APC003 | 1:100 | 3176007B | AB_2811236 | Fluidigm |
| **BM panel** | | | | | | |
| 173Yb | c-Kit | 2B8 | 1:100 | 3173004B | AB_2811230 | Fluidigm |
| 169Tm | Sca-1 | D7 | 1:100 | 3169015B | AB_2811233 | Fluidigm |
| 159Tb | F4/80 | BM8 | 1:100 | 3159009B | AB_2811238 | Fluidigm |
| 144Nd | B220 | RA3-6B2 | 1:100 | 3144011B | AB_2811239 | Fluidigm |
| 143Nd | CD11b | M1/70 | 1:100 | 3143015B | AB_2811240 | Fluidigm |
| 168Er | CD8a | 53-6.7 | 1:100 | 3168003B | AB_2811241 | Fluidigm |
| 172Yb | CD4 | RM4-5 | 1:100 | 3172003B | AB_2811242 | Fluidigm |
| 147Sm | CD45 | 30-F11 | 1:100 | 3147003B | AB_2811243 | Fluidigm |
| 209Bi | CD11c | N418 | 1:100 | 3209005B | AB_2811244 | Fluidigm |
| 170Er | Biotin | 1D4-C5 | 1:100 | 3170003B | AB_2811234 | Fluidigm |
| 160Gd | FITC | FIT-22 | 1:100 | 3160011B | AB_2811235 | Fluidigm |
| 176Yb | APC | APC003 | 1:100 | 3176007B | AB_2811236 | Fluidigm |
| **Intracellular protein panel** | | | | | | |
| 156Gd | p-p38 [T180/Y182] | D3F9 | 1:100 | 3156002A | AB_2661826 | Fluidigm |
| 152Sm | pAkt [S473] | D9E | 1:50 | 3152005A | AB_2811246 | Fluidigm |
| 158Gd | pStat3 [Y705] | 4/P-Stat3 | 1:50 | 3158005A | AB_2811100 | Fluidigm |
| 165Ho | β-Catenin | D13A1 | 1:100 | 3165027A | AB_2811247 | Fluidigm |
| 153Eu | pStat1 [Y701] | 58D6 | 1:50 | 3153003A | AB_2811248 | Fluidigm |
| 164Dy | IκBα | L35A5 | 1:100 | 3164004A | AB_2811249 | Fluidigm |
| 171Yb | pERK 1/2 [T202/Y204] | D13.14.4E | 1:100 | 3171010A | AB_2811250 | Fluidigm |
| 175Lu | pS6 [S235/S236] | N7-548 | 1:100 | 3175009A | AB_2811251 | Fluidigm |
| 141Pr | pSHP2 [Y580] | D66F10 | 1:50 | 3141002A | AB_2811252 | Fluidigm |
| 146Nd | pEGFR [Y1068] | D7A5 | 1:100 | 3146007A | AB_2811253 | Fluidigm |
| 150Nd | pRb[S807/811] | J112-906 | 1:100 | 3150013A | AB_2811254 | Fluidigm |

Table S2. Cell number in each SPADE node of each sample

|  | Day 0 | | | | Day 12 | | | | Day 16 | | | | Day 20 | | | |
| --- | --- | --- | --- | --- | --- | --- | --- | --- | --- | --- | --- | --- | --- | --- | --- | --- |
| Node  ID | #1 | #2 | #3 | #4 | #5 | #6 | #7 | #8 | #9 | #10 | #11 | #12 | #13 | #14 | #15 | #16 |
| 1 | 1060 | 528 | 914 | 707 | 476 | 263 | 182 | 133 | 1136 | 978 | 1260 | 928 | 768 | 588 | 1108 | 861 |
| 2 | 225 | 127 | 226 | 105 | 26 | 28 | 6 | 2 | 68 | 96 | 101 | 62 | 102 | 42 | 113 | 68 |
| 3 | 206 | 67 | 174 | 97 | 52 | 57 | 29 | 18 | 251 | 216 | 255 | 170 | 192 | 108 | 258 | 212 |
| 4 | 29 | 5 | 21 | 20 | 2 | 7 | 0 | 1 | 15 | 39 | 24 | 28 | 9 | 5 | 18 | 6 |
| 5 | 5 | 1 | 0 | 6 | 118 | 12 | 8 | 36 | 42 | 44 | 54 | 31 | 47 | 37 | 56 | 58 |
| 6 | 59 | 17 | 34 | 23 | 9 | 13 | 7 | 1 | 31 | 55 | 56 | 50 | 22 | 5 | 29 | 22 |
| 7 | 161 | 52 | 126 | 96 | 19 | 18 | 9 | 9 | 97 | 122 | 90 | 88 | 89 | 38 | 117 | 59 |
| 8 | 180 | 90 | 120 | 136 | 94 | 81 | 41 | 52 | 209 | 275 | 335 | 320 | 120 | 84 | 190 | 146 |
| 9 | 28 | 13 | 15 | 23 | 185 | 47 | 23 | 45 | 139 | 113 | 139 | 122 | 117 | 79 | 161 | 142 |
| 10 | 36 | 10 | 19 | 28 | 17 | 17 | 7 | 7 | 34 | 63 | 66 | 61 | 28 | 18 | 36 | 41 |
| 11 | 15 | 10 | 11 | 11 | 2 | 6 | 1 | 1 | 16 | 38 | 28 | 38 | 14 | 2 | 14 | 17 |
| 12 | 10 | 5 | 14 | 12 | 12 | 23 | 6 | 20 | 15 | 31 | 71 | 51 | 6 | 10 | 14 | 16 |
| 13 | 244 | 146 | 258 | 96 | 35 | 31 | 2 | 4 | 83 | 118 | 82 | 81 | 101 | 43 | 95 | 58 |
| 14 | 78 | 34 | 86 | 27 | 7 | 5 | 0 | 1 | 17 | 32 | 19 | 14 | 18 | 11 | 26 | 11 |
| 15 | 32 | 11 | 49 | 26 | 0 | 0 | 0 | 0 | 8 | 5 | 7 | 5 | 68 | 5 | 39 | 12 |
| 16 | 21 | 11 | 13 | 15 | 7 | 4 | 0 | 2 | 27 | 27 | 32 | 32 | 30 | 7 | 25 | 17 |
| 17 | 215 | 118 | 172 | 86 | 56 | 27 | 2 | 3 | 71 | 101 | 82 | 78 | 60 | 27 | 63 | 49 |
| 18 | 204 | 86 | 192 | 174 | 264 | 133 | 106 | 123 | 492 | 400 | 541 | 408 | 325 | 222 | 434 | 371 |
| 19 | 126 | 52 | 87 | 57 | 16 | 16 | 7 | 3 | 49 | 74 | 44 | 44 | 46 | 10 | 55 | 27 |
| 20 | 44 | 16 | 42 | 29 | 3 | 10 | 1 | 0 | 23 | 32 | 15 | 13 | 18 | 4 | 25 | 10 |
| 21 | 174 | 66 | 115 | 90 | 32 | 35 | 5 | 9 | 78 | 154 | 129 | 131 | 68 | 34 | 108 | 72 |
| 22 | 50 | 21 | 32 | 25 | 6 | 5 | 0 | 0 | 10 | 34 | 34 | 25 | 15 | 6 | 14 | 12 |
| 23 | 176 | 86 | 174 | 114 | 27 | 27 | 14 | 6 | 179 | 119 | 126 | 74 | 124 | 72 | 183 | 100 |
| 24 | 62 | 20 | 43 | 27 | 2 | 12 | 1 | 0 | 11 | 25 | 17 | 6 | 18 | 7 | 19 | 9 |
| 25 | 106 | 40 | 80 | 44 | 12 | 3 | 1 | 1 | 25 | 30 | 27 | 27 | 40 | 15 | 28 | 9 |
| 26 | 101 | 43 | 77 | 58 | 10 | 11 | 8 | 8 | 59 | 81 | 100 | 92 | 54 | 21 | 84 | 51 |
| 27 | 11 | 8 | 23 | 14 | 0 | 1 | 0 | 0 | 2 | 2 | 4 | 1 | 3 | 1 | 8 | 8 |
| 28 | 39 | 12 | 23 | 15 | 35 | 20 | 17 | 12 | 123 | 53 | 101 | 60 | 74 | 52 | 114 | 62 |
| 29 | 43 | 12 | 34 | 24 | 17 | 10 | 3 | 4 | 39 | 28 | 44 | 33 | 27 | 24 | 56 | 32 |
| 30 | 18 | 7 | 14 | 6 | 11 | 9 | 4 | 7 | 41 | 37 | 52 | 45 | 20 | 17 | 36 | 28 |
| 31 | 10 | 3 | 2 | 7 | 128 | 20 | 10 | 34 | 73 | 44 | 58 | 28 | 53 | 36 | 72 | 75 |
| 32 | 50 | 26 | 33 | 48 | 75 | 57 | 31 | 52 | 110 | 142 | 233 | 213 | 56 | 47 | 96 | 85 |
| 33 | 16 | 7 | 9 | 12 | 26 | 10 | 7 | 8 | 44 | 26 | 40 | 20 | 22 | 20 | 54 | 23 |
| 34 | 320 | 146 | 231 | 167 | 61 | 40 | 15 | 16 | 180 | 186 | 165 | 138 | 162 | 69 | 230 | 119 |
| 35 | 22 | 12 | 26 | 18 | 88 | 34 | 29 | 39 | 86 | 92 | 127 | 119 | 52 | 30 | 84 | 56 |
| 36 | 31 | 15 | 35 | 31 | 10 | 14 | 5 | 6 | 58 | 69 | 68 | 70 | 44 | 14 | 45 | 33 |
| 37 | 7 | 3 | 1 | 3 | 47 | 12 | 5 | 26 | 15 | 29 | 27 | 27 | 15 | 11 | 24 | 26 |
| 38 | 84 | 38 | 76 | 61 | 10 | 18 | 5 | 4 | 58 | 47 | 61 | 38 | 38 | 15 | 52 | 43 |
| 39 | 48 | 21 | 34 | 31 | 3 | 9 | 1 | 3 | 20 | 32 | 28 | 25 | 13 | 10 | 23 | 16 |
| 40 | 25 | 6 | 26 | 12 | 12 | 14 | 4 | 5 | 34 | 47 | 61 | 40 | 23 | 12 | 34 | 19 |
| 41 | 412 | 242 | 347 | 214 | 109 | 90 | 21 | 12 | 215 | 267 | 260 | 207 | 144 | 128 | 221 | 146 |
| 42 | 39 | 13 | 24 | 23 | 34 | 6 | 4 | 7 | 74 | 45 | 61 | 47 | 42 | 23 | 78 | 42 |
| 43 | 6 | 0 | 5 | 1 | 37 | 19 | 9 | 25 | 15 | 38 | 34 | 56 | 9 | 8 | 19 | 27 |
| 44 | 30 | 15 | 18 | 7 | 10 | 9 | 3 | 2 | 6 | 33 | 38 | 48 | 11 | 6 | 12 | 8 |
| 45 | 74 | 29 | 38 | 50 | 239 | 76 | 63 | 67 | 229 | 136 | 201 | 151 | 134 | 130 | 220 | 183 |
| 46 | 2 | 0 | 5 | 6 | 14 | 9 | 6 | 22 | 12 | 22 | 23 | 45 | 11 | 9 | 11 | 9 |
| 47 | 6 | 3 | 5 | 3 | 10 | 2 | 3 | 9 | 7 | 18 | 32 | 29 | 8 | 3 | 11 | 9 |
| 48 | 2 | 2 | 2 | 0 | 18 | 4 | 2 | 9 | 7 | 19 | 8 | 10 | 2 | 3 | 14 | 11 |
| 49 | 6 | 1 | 1 | 5 | 21 | 8 | 4 | 5 | 25 | 28 | 24 | 35 | 22 | 5 | 24 | 12 |
| 50 | 2 | 0 | 0 | 0 | 44 | 4 | 2 | 5 | 16 | 7 | 17 | 5 | 9 | 10 | 19 | 26 |
